# Transcription factor CsTT8 promotes fruit coloration by positively regulating the methylerythritol 4-phosphate pathway and carotenoid biosynthesis pathway in citrus (*Citrus* spp.)

**DOI:** 10.1093/hr/uhad199

**Published:** 2023-10-10

**Authors:** Quan Sun, Zhengchen He, Ranran Wei, Yingzi Yin, Junli Ye, Lijun Chai, Zongzhou Xie, Wenwu Guo, Juan Xu, Yunjiang Cheng, Qiang Xu, Xiuxin Deng

**Affiliations:** National Key Laboratory for Germplasm Innovation and Utilization of Horticultural Crops, Huazhong Agricultural University, Wuhan 430070, China; National Research Center for Apple Engineering and Technology, Shandong Agricultural University, Taian, Shandong 271018, China; National Key Laboratory for Germplasm Innovation and Utilization of Horticultural Crops, Huazhong Agricultural University, Wuhan 430070, China; National Key Laboratory for Germplasm Innovation and Utilization of Horticultural Crops, Huazhong Agricultural University, Wuhan 430070, China; National Key Laboratory for Germplasm Innovation and Utilization of Horticultural Crops, Huazhong Agricultural University, Wuhan 430070, China; National Key Laboratory for Germplasm Innovation and Utilization of Horticultural Crops, Huazhong Agricultural University, Wuhan 430070, China; National Key Laboratory for Germplasm Innovation and Utilization of Horticultural Crops, Huazhong Agricultural University, Wuhan 430070, China; National Key Laboratory for Germplasm Innovation and Utilization of Horticultural Crops, Huazhong Agricultural University, Wuhan 430070, China; National Key Laboratory for Germplasm Innovation and Utilization of Horticultural Crops, Huazhong Agricultural University, Wuhan 430070, China; National Key Laboratory for Germplasm Innovation and Utilization of Horticultural Crops, Huazhong Agricultural University, Wuhan 430070, China; National Key Laboratory for Germplasm Innovation and Utilization of Horticultural Crops, Huazhong Agricultural University, Wuhan 430070, China; National Key Laboratory for Germplasm Innovation and Utilization of Horticultural Crops, Huazhong Agricultural University, Wuhan 430070, China; National Key Laboratory for Germplasm Innovation and Utilization of Horticultural Crops, Huazhong Agricultural University, Wuhan 430070, China; Hubei Hongshan Laboratory Wuhan, Hubei 430070, China

## Abstract

Carotenoids directly influence citrus fruit color and nutritional value, which is critical to consumer acceptance. Elucidating the potential molecular mechanism underlying carotenoid metabolism is of great importance for improving fruit quality. Despite the well-established carotenoid biosynthetic pathways, the molecular regulatory mechanism underlying carotenoid metabolism remains poorly understood. Our previous studies have reported that the Myc-type basic helix–loop–helix (bHLH) transcription factor (TF) regulates citrus proanthocyanidin biosynthesis. Transgenic analyses further showed that overexpression of *CsTT8* could significantly promote carotenoid accumulation in transgenic citrus calli, but its regulatory mechanism is still unclear. In the present study, we found that overexpression of *CsTT8* enhances carotenoid content in citrus fruit and calli by increasing the expression of *CsDXR*, *CsHDS*, *CsHDR*, *CsPDS*, *CsLCYE*, *CsZEP*, and *CsNCED2*, which was accompanied by changes in the contents of abscisic acid and gibberellin. The *in vitro* and *in vivo* assays indicated that CsTT8 directly bound to the promoters of *CsDXR*, *CsHDS*, and *CsHDR*, the key metabolic enzymes of the methylerythritol 4-phosphate (MEP) pathway, thus providing precursors for carotenoid biosynthesis and transcriptionally activating the expression of these three genes. In addition, CsTT8 activated the promoters of four key carotenoid biosynthesis pathway genes, *CsPDS*, *CsLCYE*, *CsZEP*, and *CsNCED2*, directly promoting carotenoid biosynthesis. This study reveals a novel network of carotenoid metabolism regulated by CsTT8. Our findings will contribute to manipulating carotenoid metabolic engineering to improve the quality of citrus fruit and other crops.

## Introduction

Citrus is one of the most important fruits around the world, bringing enormous economic value in some countries and regions [1]. Citrus fruit quality is determined by intrinsic qualities (such as sugar, acid, vitamin C, and aroma) and external qualities (such as size, shape, and color). Fruit color, as the most attractive appearance quality indicator, directly determines the esthetic properties and consumer preference for citrus fruit. Carotenoids are associated with citrus fruit color because of their diverse colors [2]. Compared with most fruits, citrus has a larger number of carotenoids, with ~115 types of carotenoid, making citrus a desirable material for the study of carotenoid metabolism [3].

Carotenoids are essential natural pigments widely distributed in nature. In addition to their rich colors, carotenoids also play vital roles in human dietary nutrition and plant physiology. In plants, carotenoids participate in multiple biological processes, such as photosynthesis, pollination, and photoprotection [4]. For the human body, carotenoids with a β-ring serve as precursors of vitamin A, and vitamin A exhibits an antioxidation function, thus contributing to visual protection and immunoenhancement [5]. Carotenoids without a β-ring are involved in preventing multiple diseases, such as cancer, neurological decline, and coronary heart disease [6]. Carotenoid metabolism pathways have already been elucidated, and some key carotenoid metabolic pathway genes have been identified in previous studies. The methylerythritol 4-phosphate (MEP) pathway is responsible for providing precursors for carotenoid biosynthesis, and it determines the total production of carotenoids. The MEP pathway is mainly controlled by deoxy-d-xylulose 5-5-phosphate synthase (DXS), deoxy-d-xylulose 5-5-phosphate reductoisomerase (DXR), 4-hydroxy-3-methylbut-2-enyl diphosphate (HDS), and 4-hydroxy-3-methylbut-2-enyl diphosphate reductase (HDR) [7–9]. Phytoene synthase (PSY) uses the precursors provided by the MEP pathway to initiate carotenoid biosynthesis, and PSY and phytoene desaturase (PDS) work as the key rate-limiting enzymes for carotenoid biosynthesis [10]. The carotenoid biosynthesis pathway branches when lycopene is synthesized. Lycopene ε-cyclase (LCYE) and lycopene β-cyclase (LCYB) control carotenoid flow to the α-branch and β-branch, respectively. In the β-branch, zeaxanthin epoxidase (ZEP) catalyzes the conversion of zeaxanthin into violaxanthin, providing precursors to 9-cis-ep oxycarotenoid dioxygenases (NCED) to synthesize ABA, an important hormone in plant development.

Carotenoid metabolism is not only controlled by various key rate-limiting enzymes, but also its metabolism is subjected to strict transcriptional regulation by transcription factors (TFs) [11]. Currently, TFs regulating carotenoid metabolism mainly belong to the MADS-box, NAC, AP2/ERF, bHLH (basic helix–loop–helix), WRKY, and MYB TF families [12–15]. These TFs directly control carotenoid biosynthesis by regulating relative enzyme levels or indirectly by regulating the MEP pathway. In tomato, SlWRKY35 upregulated the expression of *SlDXS1* to supply more precursors for carotenoid biosynthesis, finally promoting carotenoid accumulation [16]. SlMYB72 binds to the promoters of *SlPSY* and ζ-carotene isomerase (*Z-ISO*) to activate their expressions, thus directly promoting lycopene synthesis [17]. CpNAC1/2 bind to and activate the promoters of phytoene desaturase (*CpPDS*) and lycopene β-cyclase (*CpLCYB*) to promote papaya carotenoid accumulation [18, 19]. In citrus, TFs CsMADS3, CsMADS5, and CsMADS6 activate the expression of *CsPSY* to promote carotenoid biosynthesis [20–22]. Additionally, CsSGR, CsHB5, and CsERF6 positively regulate carotenoid metabolism to promote carotenoid accumulation in citrus [3, 23, 24]. Recently, transcriptional regulation of carotenoid biosynthesis has been widely studied. However, there have been few reports on simultaneously regulating the MEP and carotenoid biosynthesis pathways.

The bHLH superfamily is widely present in plants and animals. These bHLH superfamily TFs usually contain a conserved basic region and a bHLH region, and these two regions function in DNA binding and dimerization [25]. The bHLH TFs are widely involved in plant development processes such as reproduction, germination, stress response, hormone signal cross-talk, and pigment biosynthesis [25–28]. The E-box (CANNTG) has been reported to be the target-specific recognition site of bHLH TFs [29]. Many bHLH TFs have been found to participate in regulating plant carotenoid metabolism. PIF1, the first bHLH family member identified as a regulator of carotenoid metabolism, together with HY5, constitute a ‘light–dark switch’ to regulate carotenoid biosynthesis and accumulation in *Arabidopsis thaliana* [30, 31]. CpbHLH1 and CpbHLH2 activate *CpCYCB* and *CpLCYB* genes to regulate carotenoid biosynthesis in papaya [32]. The above research indicates that bHLH TFs play important roles in carotenoid biosynthesis regulation. However, few studies have been conducted to explore the participation of bHLH TFs in citrus carotenoid metabolism.

Our previous research has reported that CsTT8 participates in the process of citrus proanthocyanidin biosynthesis. Based on transgenic materials, we also found that overexpression of *CsTT8* could significantly promote carotenoid accumulation in citrus, but its regulatory mechanism is still unclear. In this study, we found that overexpression of *CsTT8* in citrus fruit and calli significantly increased the carotenoid level by upregulating the transcriptional levels of MEP pathway-related genes (*CsDXR*, *CsHDS*, and *CsHDR*) and carotenoid biosynthesis-related genes (*CsPDS*, *CsLCYE*, *CsZEP*, and *CsNCED2*). Furthermore, *in vitro* and *in vivo* assays further indicated that CsTT8 directly bound the above-mentioned target gene promoters and activated their expression. Overall, this study reveals a novel regulatory mechanism of carotenoid metabolism mediated by CsTT8.

## Results

### CsTT8 functions as a nuclear-localized Myc-type basic helix–loop–helix transcriptional activator

Sequence analysis showed that *CsTT8* contained a full-length 2085-bp coding sequence (CDS) encoding a protein with 694 amino acids. Theoretically, this protein’s molecular mass was 76.84 kDa with an isoelectric point of 4.99. We further searched the conserved domain of CsTT8 protein using the National Center for Biotechnology Information (NCBI) site and found that CsTT8 contained an Myc-type basic domain in its N-terminal and a bHLH domain in its C-terminal (Supplementary Data Fig. S1A). To understand the potential functions of CsTT8, we conducted an evolutionary tree analysis between CsTT8 and other TT8s reported in plants. The phylogenetic analysis showed that CsTT8, PpeTT8 (from *Prunus persica*), and PavTT8 (from *Prunus avium* L.) were clustered closely in the same branch (Supplementary Data Fig. S1B). Besides, the multiple sequence alignments indicated that CsTT8, PpeTT8, and PavTT8 all contained a bHLH-MYC N-terminal and a bHLH domain (Supplementary Data Fig. S1C). PpeTT8 has been reported to regulate anthocyanin biosynthesis in peach [33]. Citrus mainly accumulated carotenoids, which was different from peach. Based on this, we speculated CsTT8 might take part in the carotenoid biosynthesis process in citrus.

**Figure 1:**
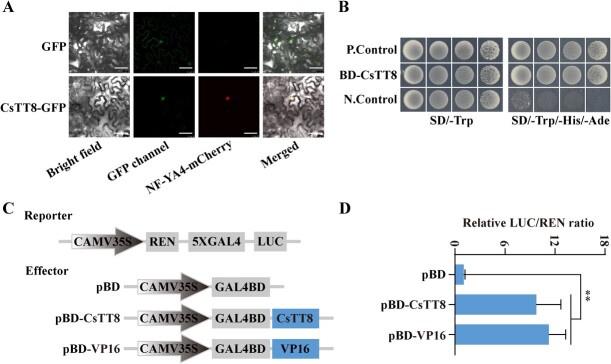
CsTT8 acts as a nucleus-localized transcriptional activator. **A** Subcellular localization analysis of CsTT8-GFP. NF-YA4-mCherry was used as a nuclear marker. CsTT8-GFP, GFP signal; NF-YA4-mCherry, RFP signal; Merged, combined GFP and RFP signals. Background colors were bright-field and white light. Bars = 50 μm. **B** Transcriptional activity analysis of CsTT8. Empty vector PGBKT7 and PGBKT7–53 + PGADT7-RecT were used as negative control (N. Control) and positive control (P. Control), respectively. **C**, **D** Transcriptional activity assays of CsTT8. pBD-VP16 and empty vector pBD served as the positive and negative control, respectively. Transcriptional activity of CsTT8 was quantified using a dual luciferase assay. Data are expressed as mean ± standard deviation of at least three biological replicates. Statistically significant differences were determined by Student’s *t*-test (^**^P < .01).

To identify the subcellular location of CsTT8, we connected *CsTT8* without the stop codon to GFP vector to construct the CsTT8-GFP (green fluorescence) fusion vector. Subsequently, this fusion vector was co-transformed with NF-YA4-mCherry (nuclear marker, red fluorescence) into *Nicotiana benthamiana* leaves. The results showed that red and green fluorescence co-existed in the nucleus, indicating that CsTT8-GFP recombinant protein was located in the nucleus (Fig. 1A).

Further, we investigated the transcription activity of CsTT8. Full-length *CsTT8* was cloned into the pGBKT7 vector to obtain the pGBKT7-CsTT8 fusion vector, and then the obtained fusion vector was transformed into yeast cells. All the transformed cells grew well on SD/−Trp selective medium, indicating the plasmids were transformed successfully. We further cultivated these cells on SD/−Trp/−His/−Ade selective medium, and found that both the pGBKT7-CsTT8 vector and the positive control (pGBKT7–53 + pGADT7-RecT) survived, but the negative control (pGBKT7 empty vector) did not, suggesting that CsTT8 had transcriptional activity (Fig. 1B). This result suggests that the CsTT8 protein has transcriptional activity. To further examine whether CsTT8 has transcriptional activation or transcriptional inhibitory activity, we connected full-length *CsTT8* to the pBD vector and obtained a pBD-CsTT8 fusion vector as the effector to conduct a dual luciferase assay (Fig. 1C). The relative LUC/REN ratio indicated that the reporter was significantly activated by pBD-CsTT8 and pBD-VP16 (positive control), but not by pBD-EV (negative control) (Fig. 1D). The above results indicated that CsTT8 functioned as a transcriptional activator.

### CsTT8 positively regulates carotenoid accumulation in citrus

**Figure 2:**
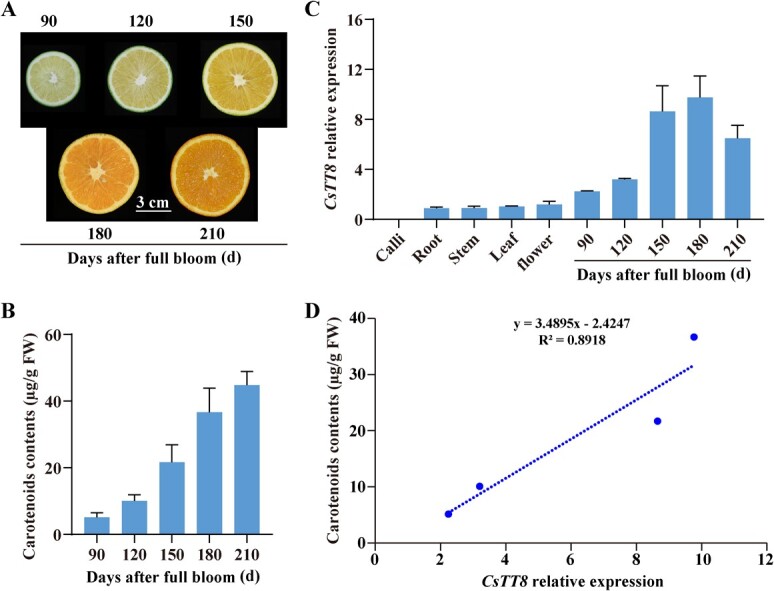
CsTT8 is closely related to fruit coloration and carotenoid biosynthesis. Changes in fruit coloration (**A**) and total carotenoid content (**B**) during ‘Valencia’ orange fruit ripening. Bars = 3 cm. **C** Temporal and spatial expression analysis of *CsTT8*. **D** Correlation analysis between *CsTT8* expression and flesh carotenoid content during citrus fruit ripening. The four data points from left to right correspond to 90, 120, 150, and 180 days after full bloom. Each data point represents the average of six biological replicates.

To explore the relationship between CsTT8 and carotenoid biosynthesis in citrus, we first tested the carotenoid contents of citrus flesh from 90–210 days after full bloom by high-performance liquid chromatography (HPLC). We found that as the flesh gradually changed from colorless to orange, its carotenoid content continued to increase (Fig. 2A and B). Subsequently, we determined the spatial and temporal expression patterns of *CsTT8* by the quantitative reverse transcription–polymerase chain reaction (qRT–PCR). The expression of *CsTT8* was lower in the root, stem, leaf, and flower than that in fruit, and it was hardly expressed in the calli (Fig. 2C). In addition, with the ripening and coloring of citrus fruits, the expression of *CsTT8* was gradually increased, reaching a maximum at 180 days after flowering, and then showed a slow decreasing trend (Fig. 2C). Correlation analysis further showed that there is a significant positive correlation between *CsTT8* expression and carotenoid content during citrus fruit ripening (Fig. 2D). The above results suggested that CsTT8 might participate in regulating carotenoid biosynthesis in citrus fruits.

**Figure 3:**
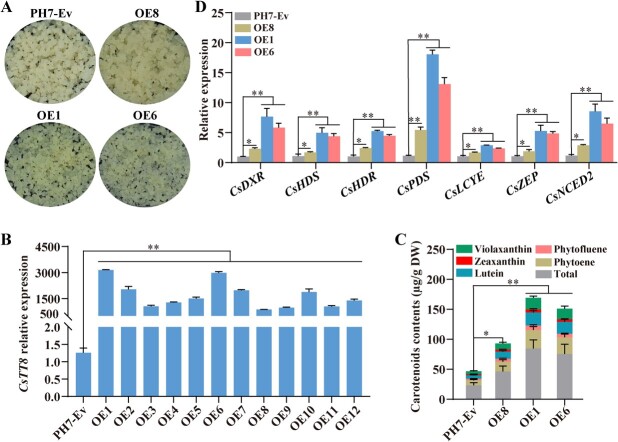
Stable overexpression of *CsTT8* promotes carotenoid accumulation in citrus calli. **A** Phenotypes of transgenic citrus calli. Empty vector PH7 was used as control (PH7-Ev). **B** Expression of *CsTT8* in *CsTT8*-overexpressing citrus calli. **C** Carotenoid content. **D** Expression of carotenoid metabolism genes. Data are expressed as mean ± standard deviation of at least three biological replicates. Statistically significant differences were determined by Student’s *t*-test (^*^P < .05; ^**^P < .01).

To further explore the function of CsTT8 in citrus carotenoid metabolism, we firstly overexpressed *CsTT8* in citrus calli stably and analyzed the carotenoid phenotype of the transgenic lines. We cloned the *CsTT8* full-length CDS into PH7WG2D overexpression vector to form the PH7-CsTT8 fusion overexpression vector, and then PH7-CsTT8 was transformed into citrus calli (Fig. 3A). Then, we detected the transcript level of *CsTT8* in positive transgenic lines by qRT–PCR, and chose the highest expression lines, OE1, OE6, and OE8, for further study (Fig. 3B). We found that citrus calli overexpressing *CsTT8* became obviously yellow compared with the control (Fig. 3A). The contents of individual compounds and the total carotenoid content of OE1, OE6, and OE8 were significantly higher than in the control (Fig. 3C). To explore the carotenoid content differences between transgenic lines and the control, we detected the expression levels of carotenoid metabolism-related genes in these transgenic calli. qRT–PCR results showed that *CsDXR*, *CsHDS*, *CsHDR*, *CsPDS*, *CsLCYE*, *CsZEP*, and *CsNCED2* were upregulated significantly in OE1, OE6, and OE8 (Fig. 3D). These results indicated that overexpression of *CsTT8* could promote carotenoid accumulation by upregulating the above carotenoid metabolism-related genes in citrus calli.

**Figure 4:**
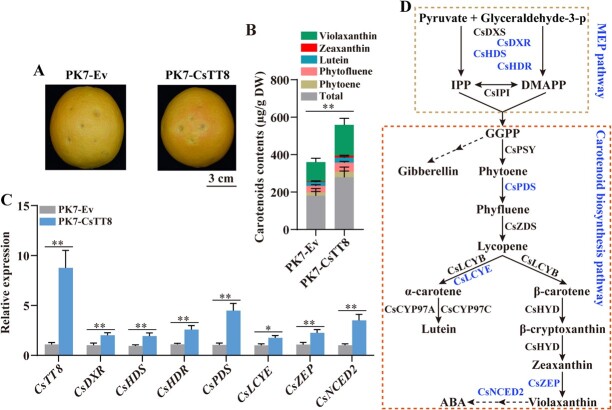
Transient overexpression of *CsTT8* promotes fruit coloration and carotenoid accumulation in citrus. **A** Citrus fruit peel coloration. PK7-Ev indicates empty vector PK7, which was used as control. PK7-CsTT8 indicates overexpression of *CsTT8*. Bar = 3 cm. **B**, **C** Carotenoid content (**B**) and relative expressions of *CsTT8* and carotenoid biosynthesis genes (**C**) in *CsTT8*-overexpressing citrus fruit. **D** Carotenoid metabolic pathway in *CsTT8*-overexpressing citrus fruit and calli. Data are expressed as mean ± standard deviation of at least three biological replicates. Statistically significant differences were determined by Student’s *t*-test (^*^P < .05; ^**^P < .01).

Subsequently, we connected *CsTT8* to PK7WG2D vector and obtained the PK7-CsTT8 fusion overexpression vector, and then injected PK7-CsTT8 into citrus peels to transiently overexpress *CsTT8* with PK7-EV used as the control (Fig. 4). The area surrounding PK7-CsTT8 injection became clearly orange, while the control was still yellow (Fig. 4A). HPLC results also showed that overexpression of *CsTT8* significantly increased total carotenoid content and individual compound contents in peel around the infiltrated sites compared with the control (Fig. 4B). We further investigated the relative expression of *CsTT8* and carotenoid biosynthesis genes in the injection area, and found that *CsTT8* was overexpressed successfully, thus leading to significant upregulation of *CsDXR*, *CsHDS*, *CsHDR*, *CsPDS*, *CsLCYE*, *CsZEP*, and *CsNCED2* compared with the control (Fig. 4C). The above results suggested that CsTT8 positively regulates citrus fruit carotenoid accumulation and fruit coloration by promoting the expression of carotenoid metabolism-related genes.

The results of overexpressing *CsTT8* stably in citrus calli and transiently expressing *CsTT8* in citrus fruit jointly indicated the effects of CsTT8 on carotenoid metabolism in citrus. As shown in Fig. 4D, overexpression of *CsTT8* activated the expression of the key rate-limiting enzyme genes *CsDXR*, *CsHDS*, and *CsHDR* in the MEP pathway. In addition, overexpression of *CsTT8* directly activated *CsPDS*, *CsLCYE*, *CsZEP*, and *CsNCED2* in the carotenoid synthesis pathway, thus directly promoting carotenoid accumulation. In summary, the overexpression of *CsTT8* upregulated the expression of the genes involved in the MEP and carotenoid biosynthesis pathways, thereby increasing the quantity of carotenoids and finally promoting carotenoid biosynthesis and accumulation in citrus.

### *CsTT8* directly activates expression of carotenoid metabolism-related genes

**Figure 5:**
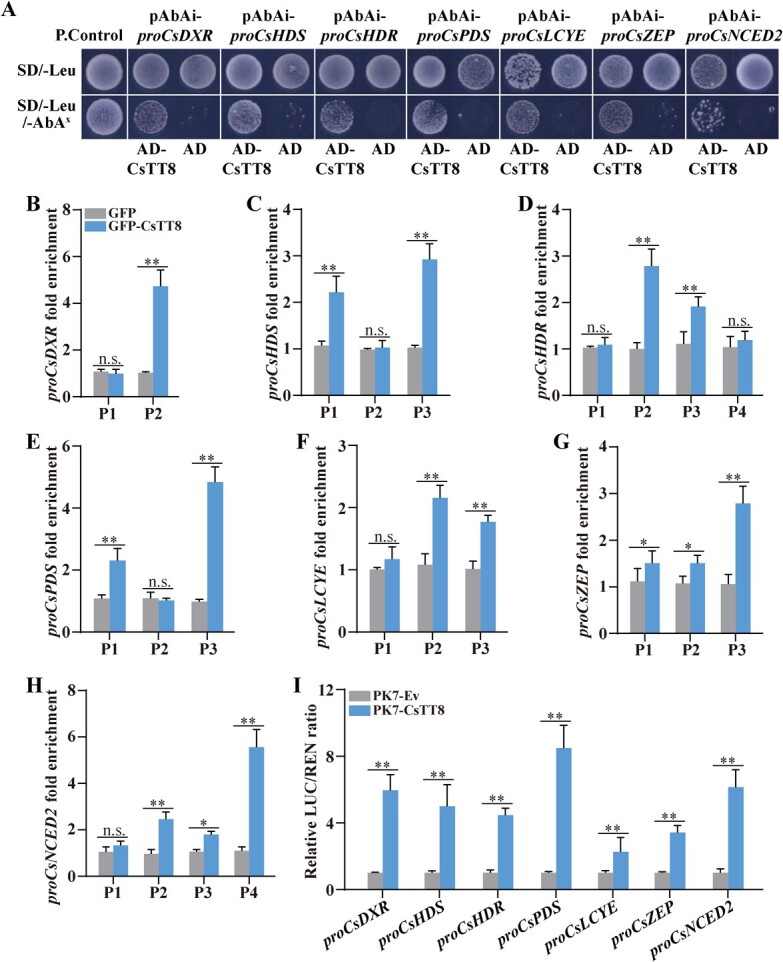
CsTT8 binds directly to promoters of key carotenoid metabolism-related genes and activates their expression. **A** Y1H assay identified interactions between CsTT8 and promoters of key carotenoid metabolic pathway genes. Empty PGADT7 + pAbAi-*proCBG* and PGADT7-Rec-p53 + p53-AbAi were used as the negative control and positive control (P. Control), respectively. AbA was used as a yeast cell growth inhibitor. SD/−Leu/AbA^x^ medium was supplemented with 200 ng ml^−1^ AbA (basal inhibition concentration of *proCsDXR*, *proCsHDS*, *proCsHDR*, *proCsPDS*, and *proCsLCYE*). SD/−Leu/AbA^x^ medium was supplemented with 150 ng ml^−1^ AbA (basal inhibition concentration of *proCsZEP* and *proCsNCED2*). **B**–**H** ChIP–PCR assays showing interaction between CsTT8 and several regions in the promoters of *CsDXR*, *CsHDS*, *CsHDR*, *CsPDS*, *CsLCYE*, *CsZEP*, and *CsNCED2*. Cross-linked chromatin was precipitated by GFP antibody and the resulting DNA fragment was determined by qPCR. (I) Relative CsTT8 activation activity of seven carotenoid metabolism pathway genes by dual-luciferase assay. Data are expressed as mean ± standard deviation of at least three biological replicates. Statistically significant differences were determined by Student’s *t*-test (^*^P < .05; ^**^P < .01).

Transgenic assays showed that overexpression of *CsTT8* significantly increased the expression levels of *CsDXR*, *CsHDS*, *CsHDR*, *CsPDS*, *CsLCYE*, *CsZEP*, and *CsNCED2* in citrus fruit and calli (Figs 3and 4). Based on this, we speculated that the above carotenoid metabolism-related genes might be the direct target genes of CsTT8, and they might be regulated by CsTT8. To verify this speculation, we used the yeast one-hybrid (Y1H) assay to detect the binding of CsTT8 to the promoters of target genes. The promoters of *CsDXR*, *CsHDS*, *CsHDR*, *CsPDS*, *CsLCYE*, *CsZEP*, and *CsNCED2* were cloned into the pAbAi vector to construct pAbAi-*proCBG* (carotenoid biosynthesis gene) fusion vectors, and then pAbAi-*proCBG* was co-transformed with pGADT7-CsTT8 into yeast cells. Aureobasidin A (AbA) was used as a bait yeast cell growth inhibitor. SD/−Leu selective medium was supplemented with 200 ng ml^−1^ AbA (basal inhibition concentration of *proCsDXR*, *proCsHDS*, *proCsHDR*, *proCsPDS*, and *proCsLCYE*). SD/−Leu selective medium was supplemented with 150 ng ml^−1^ AbA (basal inhibition concentration of *proCsZEP* and *proCsNCED2*). All co-transformed yeast cells grew well on SD/−Leu selective medium containing no AbA, implying that the co-transformation was successful (Fig. 5A). After all the co-transformed cells had been transferred to the selective medium containing the corresponding AbA concentration (SD/−Leu/−AbA^x^), the growth of the negative control was restrained completely, while other groups grew normally (Fig. 5A). These results indicate that CsTT8 directly bound to the promoters of the target genes.

The bHLH transcription family can recognize the E-box element (CANNTG sequence) of the promoter [34]. We also found several E-box elements on the promoters of target carotenoid biosynthesis genes (Supplementary Data Fig. S2A–G). Based on this, we speculated that CsTT8 might specifically recognize the E-box elements of promoters of target carotenoid biosynthesis structural genes and activate the expression of these genes. Therefore, we cloned these E-box element fragments of promoters of target genes and performed ChIP–PCR analysis. The ChIP–PCR results showed that CsTT8 was enriched in at least one E-box element of the target gene promoters (Fig. 5B–H), suggesting that CsTT8 directly bound to the E-box elements of target carotenoid metabolism-related gene promoters *in vivo*.

Further, we verified the binding of CsTT8 to the promoters of target carotenoid biosynthesis genes using the transient transformation LUC system. The full-length CDS of *CsTT8* was connected to PK7WG2D to construct the PK7-CsTT8 fusion vector as effector. *ProCBG* was cloned into the pGreen II 0800-LUC vector to form the pGreen II 0800-*proCBG* fusion vector as reporter (Supplementary Data Fig. S3). All these vectors were infiltrated into *N. benthamiana* leaves, and after 3 days the fluorescence intensity of these leaves was detected. The LUC/REN ratio showed that the activity of all target gene promoters in the *CsTT8-*overexpressing group was significantly higher than that in control group (Fig. 5I). These results indicated that CsTT8 directly activated the expression of *CsDXR*, *CsHDS*, *CsHDR*, *CsPDS*, *CsLCYE*, *CsZEP*, and *CsNCED2*.

### Working model of CsTT8-regulated carotenoid metabolism in citrus

**Figure 6:**
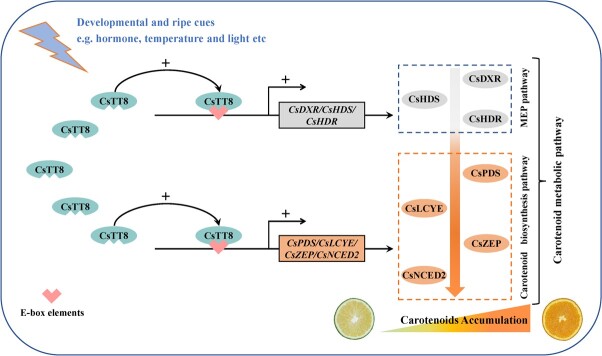
A working model of CsTT8-regulated carotenoid accumulation during citrus fruit ripening. *CsTT8* is activated by developmental and ripening cues such as hormones, temperature, and light, thus inducing the expression of key carotenoid metabolism genes (including *CsDXR*, *CsHDS*, *CsHDR*, *CsPDS*, *CsLCYE*, *CsZEP*, and *CsNCED2*), eventually promoting carotenoid accumulation and fruit coloration during citrus fruit ripening. CsDXR, 1-deoxy-d-xylulose 5-phosphate reductoisomerase; CsHDS, hydroxymethylbutenyl 4-diphosphate synthase; CsHDR, 4-hydroxy-3-methylbut-2-enyl diphosphate reductase; CsPDS, phytoene desaturase; CsLCYE, lycopene ε-cyclase; CsZEP, zeaxanthin epoxidase; CsNCED2, 9-cisepoxycarotenoid dioxygenase 2.

Based on above results of transgenic analyses and biochemical assays, we propose a working model to analyze the CsTT8-regulated carotenoid metabolism in citrus (Fig. 6). *CsTT8* is induced by a variety of developmental and ripening signals (such as hormones, temperature, and light). Subsequently, it directly binds to the promoters of MEP pathway genes (*CsDXR*, *CsHDS*, and *CsHDR*), thus increasing their expression, eventually providing sufficient substrates for the downstream carotenoid biosynthesis pathway. Meanwhile, CsTT8 significantly activates the expression of multiple rate-limiting enzyme genes (*CsPDS*, *CsLCYE*, *CsZEP*, and *CsNCED2*) involved in the carotenoid biosynthesis pathway, in turn directly promoting carotenoid accumulation. In summary, our results reveal that CsTT8 promotes carotenoid accumulation by positively regulating the MEP and carotenoid biosynthesis pathways. Our proposed working model provides valuable strategies for improving citrus fruit quality.

## Discussion

Carotenoids are important coloring pigments and secondary metabolites in nature, and they benefit human health [35, 36]. Citrus fruit accumulates different types of carotenoids during the ripening stage, thus presenting different colors. The types and contents of carotenoids determine the quality of citrus fruit, and thus carotenoids are the main factors affecting citrus fruit commodity value. Understanding the molecular regulatory mechanisms of carotenoid metabolism contribute s to the improvement of fruit quality of citrus. In this study, we elucidated a novel carotenoid metabolism regulatory network mediated by CsTT8. We found that CsTT8 promoted carotenoid accumulation by positively regulating the MEP and carotenoid biosynthesis pathways.

### CsTT8 promotes carotenoid accumulation by positively regulating the MEP pathway

The bHLH family members contain a highly conserved bHLH domain consisting of ~60 conserved amino acids, and this domain includes a basic region and a bHLH region [37]. The bHLH superfamily proteins usually participate in many physiological processes through the recognition and dimerization function of the bHLH domain [37]. Previous studies have shown that bHLH TFs are widely involved in pigment metabolism in plants [30, 32]. However, the specific molecular regulatory mechanism of bHLH TFs in citrus carotenoid metabolism remains poorly understood. In this study, *CsTT8* expression was closely correlated with citrus fruit coloration during fruit ripening (Fig. 2). Besides, overexpression of *CsTT8* led to carotenoid accumulation in citrus calli and fruit (Fig. 4C and F), which was consistent with their phenotype alteration (Fig. 4A and E). These results indicated that the Myc-type basic TF CsTT8 was involved in carotenoid metabolism during the fruit ripening process.

As is known, carotenoids are derived from two isoprene isomers, isopentenyl diphosphate (IPP) and its allylic isomer, dimethylallyl diphosphate (DMAPP). IPP and DMAPP originate from the MEP pathway in plants, which undergoes a series of condensation reactions to produce the precursor of carotenoid biosynthesis, geranylgeranyl diphosphate (GGPP) [38]. DXS, DXR, HDS, and HDR play a critical role in carotenoid metabolism as key rate-limiting enzymes for the MEP pathway [8–10]. Previous studies have demonstrated that overexpression of MEP pathway genes in *Arabidopsis* seedlings significantly promotes carotenoid accumulation [39]. Additionally, overexpression of *DXS* and *HDR* results in increased carotenoid levels in transgenic tomato fruit [40]. Therefore, carotenoid biosynthesis could be indirectly regulated by regulating the expression of *DXR*, *DXS*, and *HDR* in the MEP pathway. Although *DXR*, *DXS*, and *HDR* have been identified as important enzymes of the MEP pathway, little is known about their transcriptional regulation.

In our study, overexpression of *CsTT8* resulted in a carotenoid increase in both transgenic citrus calli and fruits, which was consistent with the significant upregulation of *CsDXR*, *CsDXS*, and *CsHDR* (Figs 3 and 4). Furthermore, Y1H, ChIP–PCR and dual-luciferase assays indicated that CsTT8 could directly bind and activate the promoters of *CsDXR*, *CsDXS*, and *CsHDR*, thus upregulating their expression levels (Fig. 5A–D and I). These results suggested that CsTT8 directly activated the expressions of *CsDXR*, *CsDXS*, and *CsHDR* in the MEP pathway, thus indirectly promoting carotenoid accumulation in citrus. Our study further confirms the function of bHLH TFs in regulating pigment metabolism, especially their involvement in plant carotenoid metabolism by directly regulating the MEP pathway.

### CsTT8 promotes carotenoid accumulation directly by regulating *CsPDS*, *CsLCYE*, *CsZEP*, and *CsNCED2* in the carotenoid biosynthesis pathway

Previous studies have shown the existence of active cross-talk between the MEP pathway and the carotenoid biosynthesis pathway [41, 42]. This cross-talk mechanism worked through the upregulation of key enzymes of the carotenoid biosynthesis pathway following the increase in carotenoid metabolism precursor, and this mechanism effectively coordinated the utilization of the precursor. *PDS*, *LCYE*, *ZEP*, and *NCED2* were crucial carotenoid metabolic genes and greatly affected carotenoid biosynthesis. CsERF061 and CsMADS6 could directly activate *CsPDS* expression to positively regulate carotenoid biosynthesis in citrus [20, 24]. A natural splicing mutation causes the inactivation of ZEP, thus leading to a sharp alteration of the carotenoid accumulation pattern, which determines orange or yellow mature fruit color in pepper [43]. These findings suggest that altering the expression pattern of the carotenoid metabolism genes can change carotenoid accumulation dramatically.

In this study, we confirmed that CsTT8 directly activated multiple MEP pathway key genes involved upstream of carotenoid metabolism to promote carotenoid accumulation indirectly in citrus. In addition, the overexpression of *CsTT8* in transgenic citrus calli and fruit also upregulated the expression of key genes in the carotenoid biosynthesis pathway (*CsPDS*, *CsLCYE*, *CsZEP*, and *CsNCED2*), involved downstream of carotenoid metabolism (Fig. 4). The results of our Y1H, ChIP-PCR, and dual-luciferase assays indicated that CsTT8 directly bound to and activated the promoter of *CsPDS*, *CsLCYE*, *CsZEP*, and *CsNCED2*, thus leading to their upregulation (Fig. 5A and E–I). The expression levels of *CsPDS*, *CsLCYE*, *CsZEP*, and *CsNCED2* were significantly upregulated in both transgenic citrus calli and fruit (Figs 3D and 4C). Activation of the MEP pathway provided more precursors for carotenoid biosynthesis. In response to the increased precursors, the expression levels of *CsPDS*, *CsLCYE*, *CsZEP*, and *CsNCED2* in the carotenoid biosynthesis pathway were upregulated to convert the precursors into more carotenoids. In line with the upregulation of *CsPDS*, *CsLCYE*, *CsZEP*, and *CsNCED2*, the total carotenoid content and the contents of individual compounds in transgenic citrus calli and fruit were increased significantly (Figs 3C and 4B). These results suggested that CsTT8 directly bound to and activated the promoters of *CsPDS*, *CsLCYE*, *CsZEP*, and *CsNCED2*, involved downstream of carotenoid metabolism, ultimately directly promoting carotenoid accumulation in citrus.

### *CsTT8* is involved in citrus ripening by regulating abscisic acid and gibberellin metabolisms

One previous study has used high-throughput RNA sequencing technology to profile *Citrus sinensis* fruit development, and the high-spatiotemporal-resolution data have shown that carotenoid and abscisic acid (ABA) biosynthesis are important processes in citrus fruit ripening [44]. Additionally, the co-expression network analysis has identified *CsTT8* as one of the hub genes of tissue-specific networks during citrus fruit ripening [44].

In our study, the overexpression of *CsTT8* resulted in increases in total carotenoid content and the contents of individual compounds in both transgenic citrus calli and fruit (Fig. 4). Furthermore, the ABA content in transgenic citrus calli and fruit was increased significantly, while the gibberellin (GA) content was decreased (Supplementary Data Fig. S4). In the carotenoid biosynthesis pathway, GGPP was the common precursor of carotenoid and GA biosynthesis. There existed a competitive relationship between GA biosynthesis and carotenoid biosynthesis. Therefore, overexpression of *CsTT8* promoted carotenoid accumulation through the activation of MEP pathway and carotenoid biosynthesis pathway, leading to more GGPP transformation to carotenoid and less GGPP transformation to GA, hence significantly decreasing GA content in transgenic citrus calli and fruit (Supplementary Data Fig. S4A and C). The increased carotenoid content provided more precursors to ABA biosynthesis. At the same time the key ABA metabolism genes, *CsZEP* and *CsNCED2*, were upregulated, thus promoting ABA biosynthesis and accumulation (Supplementary Data Fig. S4B and S4D). These results suggested that *CsTT8* participated in the regulation of ABA and GA metabolism.

Citrus is a typical non-climacteric fruit. Earlier studies regard ABA as a ripening control factor for non-climacteric fruits [45]. Interestingly, the expression of *CsTT8* increased gradually during citrus fruit ripening (Fig. 2B), and overexpression of *CsTT8* resulted in the increase in ABA content (Supplementary Data Fig. S4B, Supplementary Data Fig. S4D). These results jointly indicated that *CsTT8* might play an important role in citrus fruit ripening.

## Materials and methods

### Plant material

The citrus material ‘Valencia’ orange (*Citrus natsudaidai*) was collected at the National Citrus Breeding Center at Huazhong Agricultural University. Citrus fruits were sampled uniformly from eight directions of healthy trees. The citrus fruit peel was separated from sampled fruits and quick-frozen in liquid nitrogen and preserved at −80°C for subsequent experiments.

### Quantitative reverse transcription–polymerase chain reaction

The total RNA was extracted according to a previously reported method [46, 47]. The qRT–PCR was performed following previously reported procedures [48]. Each experiment was conducted in triplicate. The qRT–PCR primers are listed in Supplementary Data Table S1.

### Gene cloning and sequence analysis

The full-length CDS of *CsTT8* was amplified from ‘Valencia’ orange flesh by PCR based on the Citrus Pan-breeding to Genome database (http://citrus.hzau.edu.cn/). CLUSTAL W and GeneDoc softwares were used for multiple sequence alignments.

### Stable transformation of citrus calli

The full-length CDS of *CsTT8* was cloned into PH7WG2D with a GFP tag to construct the PH7-CsTT8 fusion vector. The PH7 empty vector (PH7-Ev) was used as the control. The citrus ‘RM’ callus was used in the transformation experiment according to a previously described method [47].

### Carotenoid extraction and HPLC analysis

The transgenic calli and fruit peel were ground to a powder in liquid nitrogen after full lyophilization in the lyophilizer (Labconco FreeZone^®^). Reversed-phase high-performance liquid chromatography (HPLC) was used to analyze the extracted carotenoid, as previously described [49]. Carotenoid identification was performed by comparing the characteristic spectral properties and typical retention times. The peaks of zeaxanthin, phytoene, lutein, phytofluene, and violaxanthin were at 45 0, 286, 348, 447, and 437 nm, respectively. The carotenoid content was quantified by calibration curves prepared by using the corresponding standards. The calibration curves are listed in Supplementary Data Table S1. Carotenoid standards (phytoene, phytofluene, lutein, zeaxanthin, and violaxanthin) were purchased from CaroteNature (Lupsingen, Switzerland). Each sample had at least three independent biological replicates.

### Subcellular localization of *CsTT8*

The subcellular localization of CsTT8 was determined as previously described [50]. The CDS without the stop codon was cloned and connected to pM999-35S to obtain the 35::CsTT8-GFP fusion vector. NF-YA-mCherry was used as the nuclear marker in the experiment. A confocal laser scanning microscope (TCS SP2; Leica Germany) was used for imaging the florescence images.

### Transcriptional activation assay

The full-length CDS of *CsTT8* was connected to the pGBKT7 vector to construct the pGBKT7-CsTT8 fusion vector. pGKBT7-53 + pGADT7-recT and pGBKT7 empty vector were used as the positive and negative control, respectively. All the vectors were transformed into the yeast strain AH109. The transformed yeast was cultured on selective SD/−Trp and SD/−Trp/−His/−Ade medium. The growth status of yeast cells was used to evaluate the transactivation activity of CsTT8.

The full-length CDS of *CsTT8* was connected to the pBD vector to construct the pBD-CsTT8 recombination vector. The pBD empty vector and pBD-VP16 were respectively used as negative control and positive control in the experiment. The pGreen 0800-5 × GAL4 vector was used as reporter. All the vectors were transformed into *Agrobacterium tumefaciens* strain GV3101, and then injected into *N. benthamiana* leaf, as previously described [47]. The relative LUC/REN ratio was used to evaluate the transcription activation of CsTT8.

### Yeast one-hybrid assay

The promoter fragments of *CsDXR*, *CsHDS*, *CsHDR*, *CsPDS*, *CsLCYE*, *CsZEP*, and *CsNCED2* were amplified and connected to the pABAi vector to generate the baits. The recombinant pGADT7-CsTT8 was transformed into the Y1H Gold strain containing the above baits. The Y1H assay was conducted according to the manufacturer’s protocol (Matchmaker^®^ Gold Y1H Library Screening System User Manual; TaKaRa). The growth status of transformed yeast cells was used to determine the interactions between CsTT8 protein and promoters.

### Dual luciferase reporter assay

The full-length CDS of *CsTT8* was connected to PK7WG2D to generate the PK7-CsTT8 overexpression vector. The promoters of *CsDXR*, *CsHDS*, *CsHDR*, *CsPDS*, *CsLCYE*, *CsZEP*, and *CsNCED2* were inserted into the pGreenll 0800-LUC vector to construct the reporter. The empty PK7WG2D was used as the negative control. The bacterial suspension (GV3101) was mixed with effector and reporter at a ratio of 5:1 and infiltrated into *N. benthamiana* leaves. After 3 days, the Dual-Luciferase Reporter Assay System (Promega) with an Infinite 200 Pro microplate reader (Tecan) was used for determining the luciferase activity.

### ChIP–PCR

The ChIP–PCR was conducted according to the protocol of the EpiQuik™ Plant ChIP Kit (Cat. #P-2014, Epigentek, Farmingdale, NY, USA). High-expression transgenic GFP-CsTT8 lines grown for ~15–20 days were vacuumized under formic acid for 10 min to cross-link DNA and protein. Chromatin DNA was extracted and split by ultrasound into 200- to 1000-bp pieces of DNA. One hundred microliters of DNA fragments was incubated with 5 μl of GFP antibody at room temperature for 2 h for co-precipitation, and another 100 μl of DNA fragments was incubated with mouse antibody (IgG, Epigentek) as the negative control. DNA fragments were dissociated and purified, then used for qRT–PCR analysis of relative expression levels of the fragments so as to determine the binding of *CsTT8* and promoters. Primers used in qRT–PCR are listed in Supplementary Data Table S1.

### Statistical analysis

The data were expressed as mean ± standard deviation of three independent replicates. The statistical analysis of data was performed by Microsoft Office 2010 and GraphPad 8.0 softwares.

## Supplementary Material

Revised-Supplemental_Table_S1_uhad199

Revised-Supplemental_Figure_uhad199

## Data Availability

All relevant data are included in the paper and its supplementary files. Sequence data can be found in CPBD (http://citrus.hzau.edu.cn/). All accession numbers are listed in Supplementary Data Table S1.
