# Supplementary material for: Transcription factor CsTT8 promotes fruit coloration by positively regulating the methylerythritol 4-phosphate pathway and carotenoid biosynthesis pathway in citrus (Citrus spp.)
Source: Hortic Res. 2023 Oct 10;10(11):uhad199. doi: 10.1093/hr/uhad199 (PMC10673655; doi:10.1093/hr/uhad199)
Supplement: Revised-Supplemental_Figure_uhad199 [file revised-supplemental_figure_uhad199.zip › Revised-Supplemental_Figure_uhad199.docx]

**
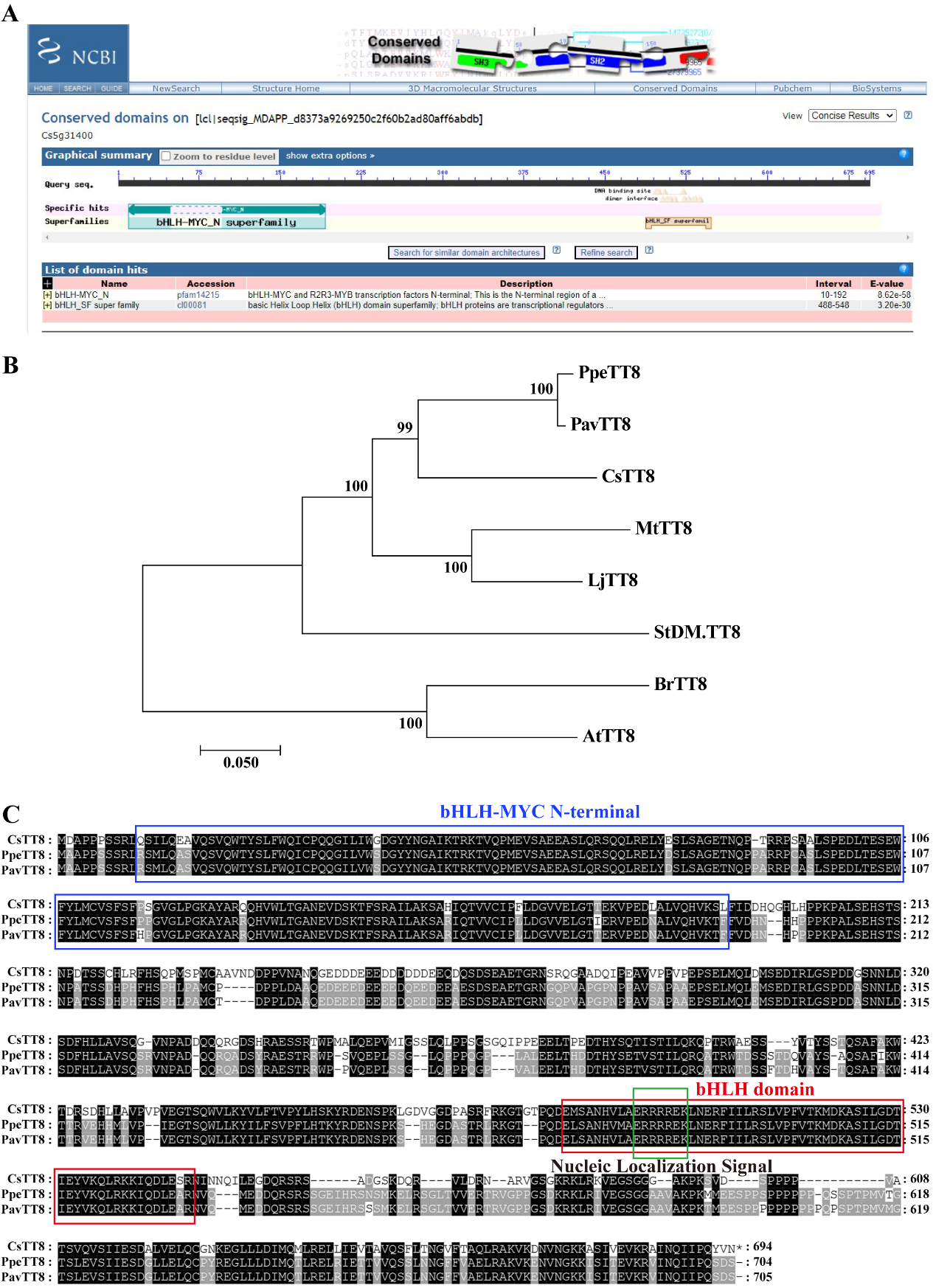
**

**Supplemental Figure 1. A novel** **Myc-type basic helix-loop-helix (bHLH) family protein, CsTT8, is identified.**

**(A)** Domain analysis of CsTT8 was conducted on the National Center for Biotechnology Information (NCBI, <https://www.ncbi.nlm.nih.gov/>). **(B)** Phylogenetic analysis of CsTT8 and other TT8 proteins from NCBI. The scale bar represents 0.05 substitutions per site. **(C)** Multiple sequence alignments of CsTT8, PpeTT8 from peach (*Prunus persica*), and PavTT8 from sweet cherry (*Prunus avium* L.).

**
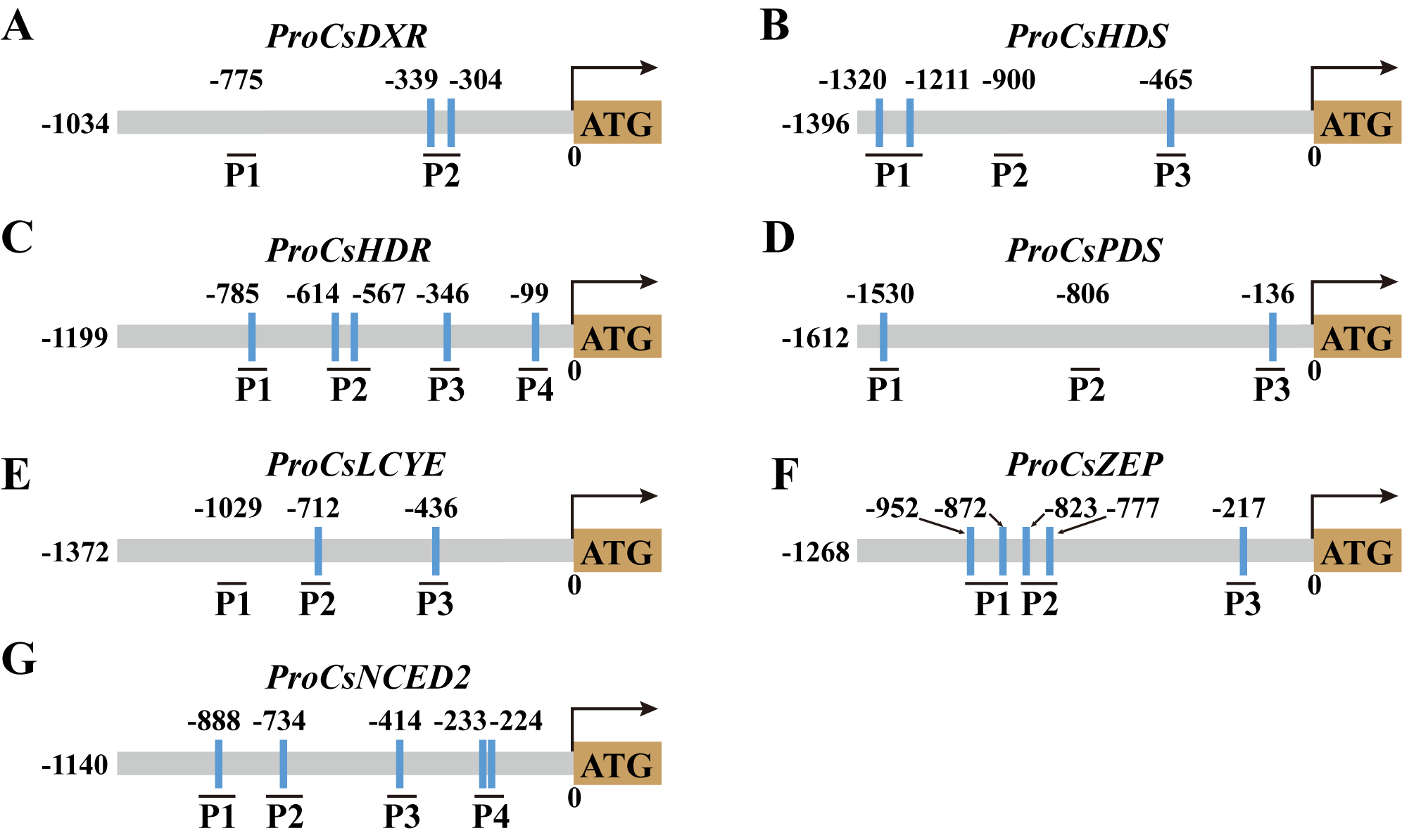
**

**Supplemental Figure 2. Schematic diagram of target gene promoters.**

(A)-(G) Schematic diagram of target gene promoters (*CsDXR*, *CsHDS*, *CsHDR*, *CsPDS*, *CsLCYE*, *CsZEP*, and *CsNCED2*). These blue lines represent putative CsTT8 binding motif of these promoters.


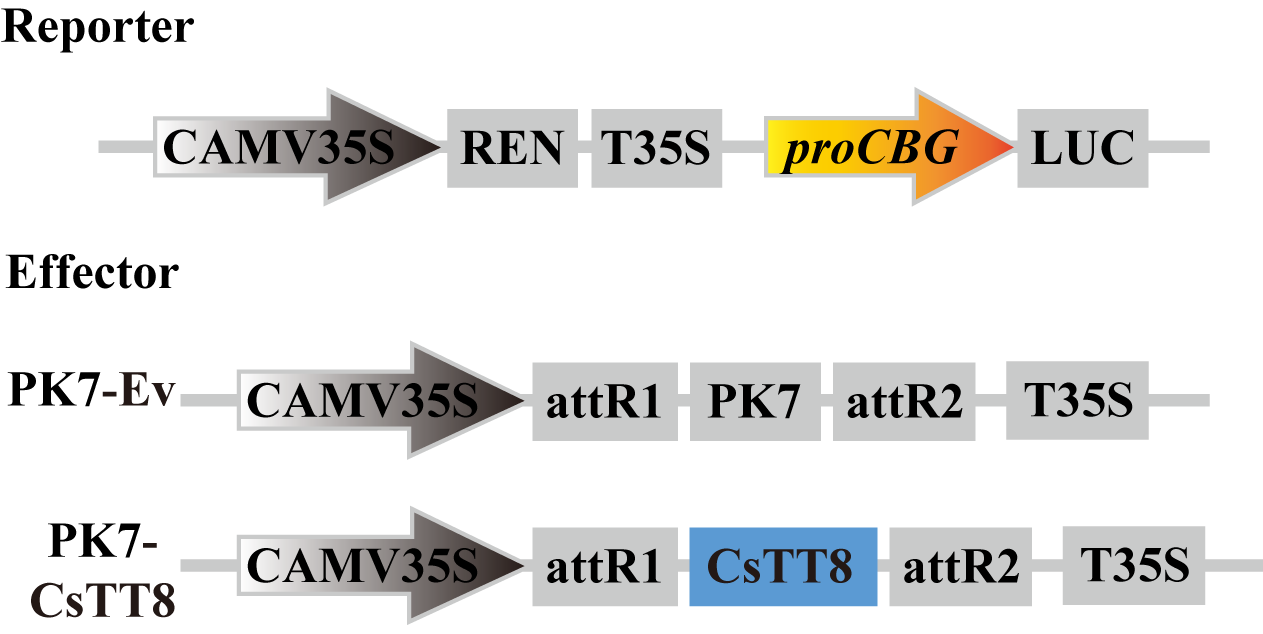


**Supplemental Figure 3. Schematic diagram of reporter and effector constructs used for the dual-luciferase assay.**

CBG indicates carotenoid biosynthesis genes.

**
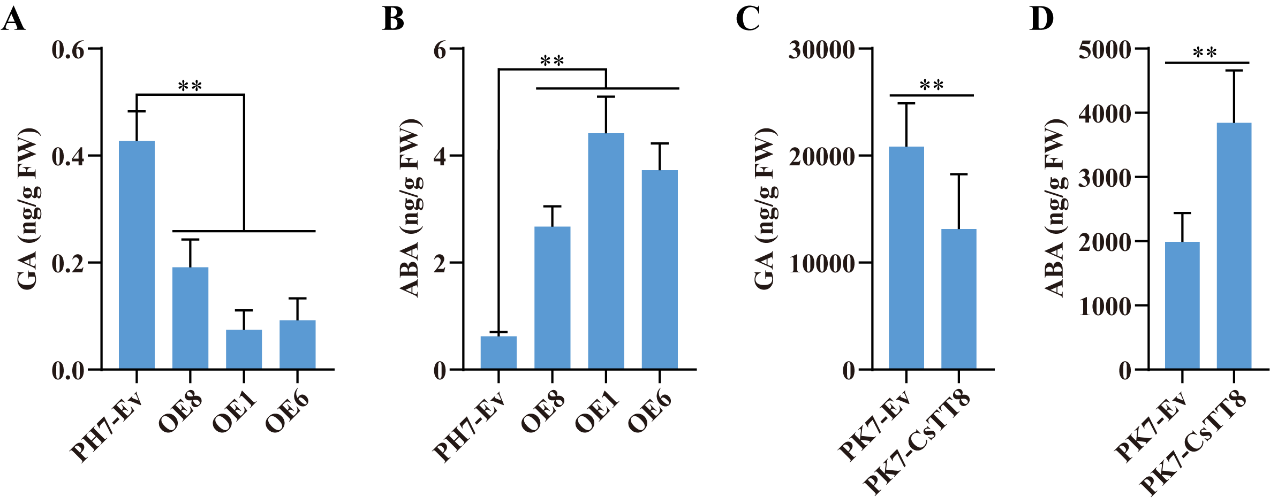
**

**Supplemental Figure 4. Effects of *CsTT8* overexpression on the content of GA and ABA in citrus.**

Changes in the GA concentration of transgenic citrus calli **(A)** and transiently injected citrus fruit **(C)** relative to the control. Changes in the ABA concentration of transgenic citrus calli **(B)** and transiently injected citrus fruit **(D)** relative to the control. The data were expressed mean ± SD of at least three biological replicates. Statistically significant differences were determined by Student’s t-test (*, *P*< 0.05; **, *P*< 0.01).
